# Supplementary material for: Valley polarization assisted spin polarization in two dimensions
Source: Nat Commun. 2015 Jun 1;6:7230. doi: 10.1038/ncomms8230 (PMC4458877; doi:10.1038/ncomms8230)
Supplement: Supplementary Information — Supplementary Figures 1-2, Supplementary Discussion and Supplementary References [file ncomms8230-s1.pdf]

SUPLEMENTARY FIGURES

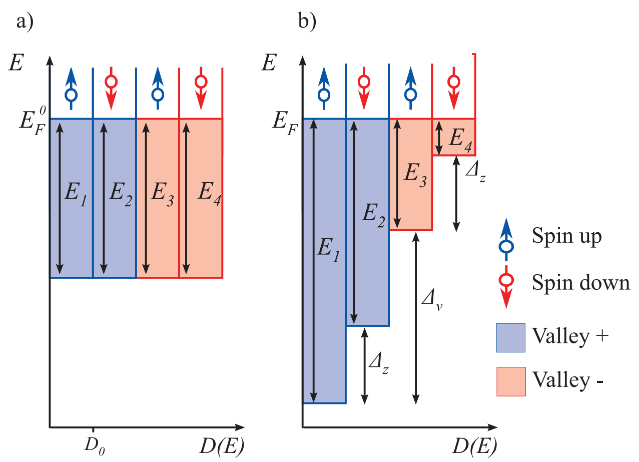

Supplementary Figure 1. **Simple energy diagram of a two-dimensional electron system**. Subbands of different valleys (+) and (-) are split by Δ_v while subbands of opposite spin are split by Δ_z. Each spin-valley split subband labeled 1 to 4 has its own Fermi energy E_1 to E_4 defined as the energy difference between the Fermi level and the subband bottom. The system is presented in absence of valley- and spin-splitting in a) while it presented in presence of valley- and spin-splitting in b).

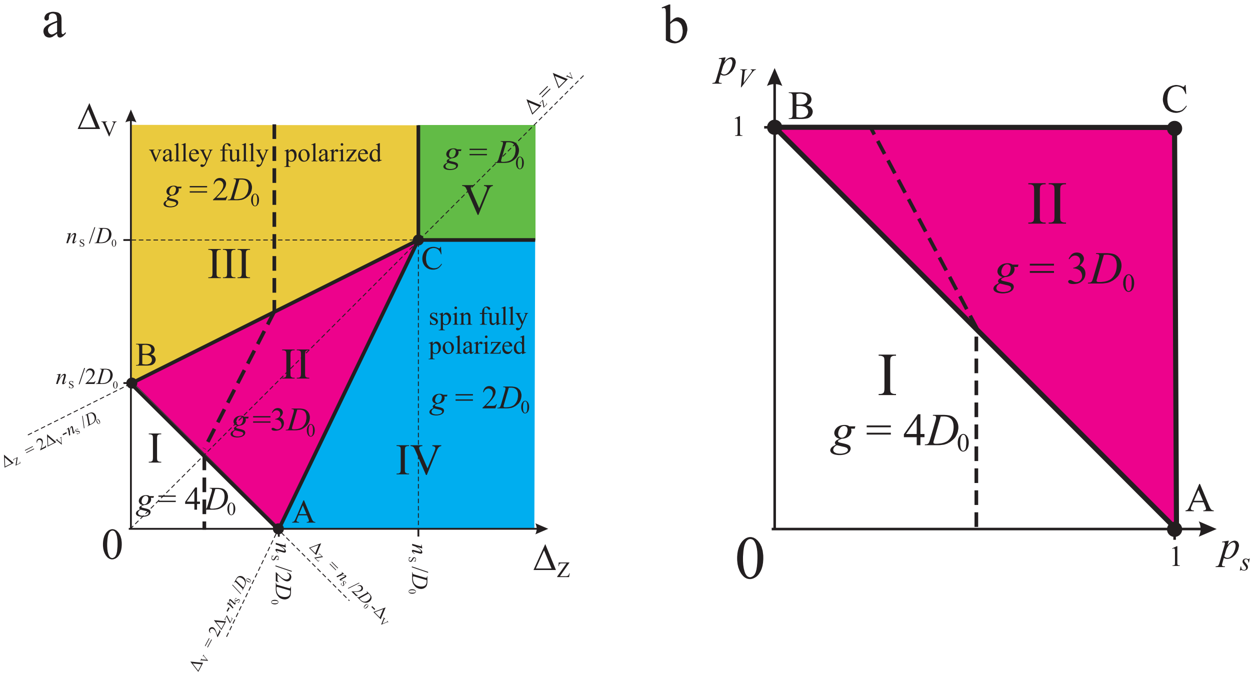

Supplementary Figure 2. **Density of states map**. Regions with different density of states g are marked I to V and key boundaries between them are marked as derived in the body text. a) Representation in the (Δ_z, Δ_v) plane. The bold dashed line represents a line of constant spin polarisation (see the text) b) Representation in the (p_z, p_v) plane. The bold dashed line represents a line of constant spin splitting (see the text).

SUPPLEMENTARY DISCUSSION: SINGLE PARTICLE PICTURE

In this section, we derive how spin and valley polarisation relate to spin and valley splitting according to the simplest single particle model.

A. Subbands

In Supplementary Figure 1 we consider a simple two-dimensional electron system at zero temperature with a parabolic dispersion and a given electron density n. In absence of spin and valley splitting (Supplementary Figure 1a), the Fermi energy E_F^0 reads:

$$E_\mathrm{F}^0 = n/g_\mathrm{s}g_\mathrm{v}D_0. \tag{1}$$

where g_s and g_v are spin and valley degeneracy respectively, m_b the band mass and $D_0 = m_b/2\pi\hbar^2$ is the constant density of states in each valley-split, spin-split subband. When the valley splitting Δ_v and spin splitting Δ_z are both small, all four spin and valley split subbands are occupied (see Supplementary Figure 1b). Each subband is occupied up to the common Fermi level, but electrons at the Fermi level in each subband have different kinetic energy or Fermi energy E_1, E_2, E_3 and E_4 which are given by:

$$\begin{aligned}
E_1 &= \frac{n}{4D_0} + \frac{1}{2}(+\Delta_z + \Delta_v);\\
E_2 &= \frac{n}{4D_0} + \frac{1}{2}(-\Delta_z + \Delta_v);\\
E_3 &= \frac{n}{4D_0} + \frac{1}{2}(+\Delta_z - \Delta_v);\\
E_4 &= \frac{n}{4D_0} + \frac{1}{2}(-\Delta_z - \Delta_v).
\end{aligned} \tag{2}$$

When valley or spin splitting is increased, this is valid up to the point where subband 4 depopulates, that is, $E_4 > 0$:

$$\frac{n}{4D_0} > \frac{1}{2}(\Delta_z + \Delta_v). \tag{3}$$

and in a parameter space with Δ_v and Δ_z as axes, the region where $g(E) = 4D_0$ (region I, Supplementary Figure 2) is bound by the line

$$\Delta_z = \frac{n}{2D_0} - \Delta_v. \tag{4}$$

We have not specified whether or not spin or valley splitting is greater than the other which implies that this is valid either way, connecting the points of valley polarisation under spin degeneracy (point B, Supplementary Figure 2), and spin polarisation under valley degeneracy [point A, Supplementary Figure 2]. Upon crossing this boundary, the density of states drops from $g = 4D_0$ to $g = 3D_0$ and there are only three subbands occupied (region II, Supplementary Figure 2). The Fermi energies for each of the subbands are

now:

$$E_1 = \frac{n}{3D_0} + \frac{1}{3}(+\Delta_v + \Delta_z);$$
$$E_2 = \frac{n}{3D_0} + \frac{1}{3}(+\Delta_v - 2\Delta_z);$$
$$E_3 = \frac{n}{3D_0} + \frac{1}{3}(-2\Delta_v + \Delta_z). \quad (5)$$

Thus with increasing Δ_z, the first subband to depopulate is subband 2, i.e. region II where $g = 3D_0$ is bound by the line (A to C on Supplementary Figure 2)

$$\Delta_v = 2\Delta_z - \frac{n}{D_0} \quad (6)$$

and similarly, if Δ_v is increased faster than Δ_z, a subband is emptied along the line (B to C on Supplementary Figure 2)

$$\Delta_z = 2\Delta_v - \frac{n}{D_0}. \quad (7)$$

On the (Δ_z, Δ_v) plane, these boundaries lead to two regions where only two subbands are occupied and the density of states is $g = 2D_0$. One region corresponds to full valley polarisation while spin is degenerate or only partially polarized [region III], while the other region corresponds to full spin polarisation with valley degeneracy or partial valley polarisation [region IV]. A departure from point A corresponds to valley splitting of a spin subband edge, while a departure from B corresponds to spin splitting of the valley subband edge[1,2].

When valleys are fully polarized, subbands 3 and 4 are completely empty:

$$E_1 = \frac{n}{2D_0} + \frac{1}{2}\Delta_z;$$
$$E_2 = \frac{n}{2D_0} - \frac{1}{2}\Delta_z \quad (8)$$

and the Fermi energies no longer depend on the valley splitting. Similarly, when we have full spin polarisation, subbands 2 and 4 are empty:

$$E_1 = \frac{n}{2D_0} + \frac{1}{2}\Delta_v;$$
$$E_3 = \frac{n}{2D_0} - \frac{1}{2}\Delta_v \quad (9)$$

and the Fermi energies no longer depend on the spin splitting. Further, at complete spin and valley polarisation [region V], the Fermi energy ceases to depend on either the spin or valley splitting, leading to the condition described by Supplementary Equation 1 but with $g_\mathrm{s} = g_\mathrm{v} = 1$.

B. Polarisation and Susceptibility

It is often useful to define polarisation as we have done in the main text:

$$p_\mathrm{s} = \frac{n_\uparrow - n_\downarrow}{n_\uparrow + n_\downarrow};$$
$$p_\mathrm{v} = \frac{n_+ - n_-}{n_+ + n_-} \quad (10)$$

where $n_\uparrow$ and $n_\downarrow$ are densities of electrons with up and down spin respectively while n_+ and n_- are densities of electrons in two respective valleys. For comparison, we map the regions of the $(\Delta_z,\ \Delta_v)$ parameter

space to ($p_{\rm s}$, $p_{\rm v}$). Full valley polarisation (region III in Supplementary Figure 2a) maps to the line BC in Supplementary Figure 2b and full spin polarisation (region IV in Supplementary Figure 2a) maps to the line AC in Supplementary Figure 2b. Full valley and full spin polarisation (region V Supplementary Figure 2a) maps to the point C on the ($p_{\rm s}$,$p_{\rm v}$) plane. Two regions of equal area remain corresponding to I ($g = 4D_0$) where all four subbands are occupied and II (($g = 3D_0$)) where one subband has been lifted above the Fermi level. These two regions are separated by

$$p_{\rm v} + p_{\rm s} = 1. \tag{11}$$

Spin and valley polarisation is achieved by applying symmetry breaking field. We now consider how polarisation is altered by these fields, i.e. their susceptibilities. Spin and valley susceptibilities can be defined:

$$\chi_s = \frac{\partial p_{\rm s}}{\partial B};$$
$$\chi_v = \frac{\partial p_{\rm s}}{\partial F} \tag{12}$$

where B is the magnetic flux density and F is a valley-field which acts to increase valley splitting. In the case of AlAs structures[3–5], F would correspond to the strain, ϵ, while in our SIMOX based silicon devices[6,7], it corresponds to δn, which quantifies the out-of-plane potential asymmetry (See Section III).

In region I of Supplementary Figure 2a when all four subbands are occupied, $n_\uparrow = D_0(E_1 + E_3)$ and $n_\downarrow = D_0(E_2 + E_4)$. It therefore follows that:

$$p_{\rm s} = 2\Delta_z \frac{D_0}{n};$$
$$\chi_s = 2D_0 \frac{\partial \Delta_z}{\partial B} = \frac{m_b g \mu_{\rm B}}{n\pi\hbar^2} \tag{13}$$

which is the well-known result for a two-fold valley degenerate 2DEG. In region II, when one subband is lifted up beyond the Fermi level, the spin polarisation becomes

$$p_{\rm s} = \frac{1}{3} + \frac{D_0}{3n}(4\Delta_z - 2\Delta_v) \tag{14}$$

which clearly obtains a dependence on valley splitting which was absent in the case where all four subbands were occupied. Also, the susceptibility becomes

$$\chi_s = \frac{4D_0}{3n}\frac{\partial \Delta_z}{\partial B} = \frac{2}{3}\frac{m_b g \mu_{\rm B}}{n\pi\hbar^2} \tag{15}$$

which is a significant reduction compared with region I. In region III, the system is fully valley polarized and the spin polarisation and susceptibility regain their usual expressions for non-valley-degenerate systems:

$$p_{\rm s} = \frac{\Delta_z D_0}{n};$$
$$\chi_s = D_0 \frac{\partial \Delta_z}{\partial B} = \frac{m_b g \mu_{\rm B}}{2n\pi\hbar^2}. \tag{16}$$

In regions IV and V, spin is already fully polarized and the susceptibility is zero. Clearly, by symmetry, similar expressions can be written for valley polarisation and valley susceptibility.

It is important to point out that when considering the physics of the entire parameter space, the energies Δ_v and Δ_z are not interchangeable with polarisations $p_{\rm v}$ and $p_{\rm s}$, especially in region II. We spell out the details below, although it repeats some equations we have stated already. In Region I, the two sets are

simply related by

$$p_{\rm v} = 2\Delta_v \frac{D_0}{n};$$
$$p_{\rm s} = 2\Delta_z \frac{D_0}{n}, \tag{17}$$

but in Region II, these become

$$p_{\rm v} = \frac{1}{3} + \frac{1}{3}\frac{D_0}{n_S}(-2\Delta_z + 4\Delta_v);$$
$$p_{\rm s} = \frac{1}{3} + \frac{1}{3}\frac{D_0}{n_S}(+4\Delta_z - 2\Delta_v). \tag{18}$$

In Region III

$$p_{\rm v} = 1;$$
$$p_{\rm s} = \frac{\Delta_z D_0}{n_S}, \tag{19}$$

and similarly in Region IV,

$$p_{\rm v} = \frac{\Delta_v D_0}{n_S};$$
$$p_{\rm s} = 1, \tag{20}$$

while in Region V, $p_{\rm v} = 1$ and $p_{\rm s} = 1$.

These equations can be used to calculate the polarisations $p_{\rm v}$ and $p_{\rm s}$ for any Δ_v and Δ_z. The implication of the fact that Supplementary Equations 18 for region II both contain Δ_v and Δ_z is that changing the energy of one degree of freedom alters the polarisation of the other. For example, starting at point A in Fig. S2, fixing $\Delta_z = n/2D_0$, $p_{\rm s}$ is initially equal to one. However, if we increase Δ_v and move up through region II, by the time we reach the boundary to region III midway on line BC, $p_{\rm s}$ has decreased by a half to $p_{\rm s} = 1/2$. Thus, valley polarisation has induced a spin depolarisation. In fact, lines of constant $p_{\rm s}$ in region II on the (Δ_v, Δ_z) plane describe lines parallel to AC and similarly, lines of constant $p_{\rm v}$ describe lines parallel to BC. An example is shown in Supplementary Figure 2a. Similarly, lines of constant energy splitting do not simply run vertically or horizontally on the polarisation plane as illustrated in Supplementary Figure 2b. As the example shows, if Δ_z is fixed, in Region II, polarizing valleys reduces the spin polarisation by a factor of two.

To conclude this section, we stress that although Eqs. 17-20 were derived in a single particle picture, they can still be used for interacting systems within a Fermi-liquid approach. In this case, density of states, Zeeman and valley gaps are renormalized by interactions. The values of $p_{\rm v}$ shown in Fig. 2 of the main manuscript were calculated using Supplementary Equations 20 and the experimentally measured values of valley gaps[7] which therefore include these effects.

---

[1] Niida Y., Takashina K., Fujiwara A., Fujisawa T. and Hirayama Y. Spin splitting of upper electron subbands in a SiO_2/Si(100)/SiO_2 quantum well with in-plane magnetic field. *Appl. Phys. Lett.*, **94**, 142101 (2009)

[2] Takashina, K. *et al.* Impact of Valley Polarization on the Resistivity in Two Dimensions. *Phys. Rev. Lett.* **106**, 196403 (2011).

[3] Shkolnikov, Y., P., Vakili, K., De Poortere, E. P., & Shayegan M. Dependence of Spin Susceptibility of a Two-Dimensional Electron System on the Valley Degree of Freedom. *Phys. Rev. Lett.* **92**, 246804 (2004).
[4] Gunawan, O. *et al.* Spinvalley phase diagram of the two-dimensional metalinsulator transition. *Nature Physics* **3**, 388 - 391 (2007)
[5] Gokmen, T. Padmanabhan, M., & Shayegan, M. Transference of transport anisotropy to composite fermions *Nature Phys.* **6**, 621624 (2010).
[6] Takashina K., Fujiwara A., Horiguchi S., Takahashi Y. & Hirayama Y., Valley splitting control in SiO_2/Si/SiO_2 quantum wells in the quantum Hall regime *Phys. Rev. B* **69**, 161304(R) (2004)
[7] Takashina K., Ono Y., Fujiwara A., Takahashi Y. & Hirayama Y. Valley Polarization in Si(100) at Zero Magnetic Field *Phys. Rev. Lett.* **96**, 236801 (2006).
